# Optimal Image Gain Intensity of Point-of-care Ultrasound when Screening for Ocular Abnormalities in the Emergency Department

**DOI:** 10.5811/westjem.59714

**Published:** 2023-05-05

**Authors:** Melissa Chang, Nicole Finney, Jessa Baker, Jonathan Rowland, Shreya Gupta, Reem Sarsour, Soheil Saadat, John C. Fox

**Affiliations:** * University of California, Irvine School of Medicine, Irvine, California; † University of California, Irvine, Department of Emergency Medicine, Irvine, California

## Abstract

**Introduction:**

Point-of-care ultrasound (POCUS) plays a pivotal role in evaluating ocular complaints in the emergency department (ED). The rapid and non-invasive nature of ocular POCUS makes it a safe and informative imaging modality. Previous studies have investigated using ocular POCUS to diagnose posterior vitreous detachment (PVD), vitreous hemorrhage (VH), and retinal detachment (RD); however, there are few studies that assess image optimization techniques and how they impact the overall accuracy of ocular POCUS.

**Methods:**

We performed a retrospective review of ED patients who received ocular POCUS examinations and ophthalmology consultations as part of their evaluation for eye complaints at our urban, Level I trauma center ED from November 2017–January 2021. Of 706 exams, 383 qualified for the study. In this study we primarily investigated how stratified gain levels impact the accuracy of ocular POCUS for detection of any posterior chamber pathology and, secondarily, whether stratified gain levels impact the accuracy of detecting RD, VH, and PVD specifically.

**Results:**

The images were found to have an overall sensitivity of 81% (76–86%), specificity of 82% (76–88%), positive predictive value (PPV) of 86% (81–91%), and negative predictive value (NPV) of 77% (70–83%). Images acquired with a gain of (25, 50] had a sensitivity of 71% (61–80%), specificity of 95% (85–99%), PPV of 96% (88–99%), and NPV of 68% (56–78%). Images acquired with a gain of (50, 75] had a sensitivity of 85% (73–93%), specificity of 85% (72–93%), PPV of 86% (75–94%), and NPV of 83% (70–92%). Images acquired with a high gain (75, 100] had a sensitivity of 91% (82–97%), specificity of 67% (53–79%), PPV of 78% (68–86%), and NPV of 86% (72–95%).

**Conclusion:**

In the ED setting, high (75, 100] gain on ocular POCUS scanning has a higher degree of sensitivity for detecting any posterior chamber abnormality, as compared to low (25, 50] gain levels. Thus, incorporating the use of high gain for ocular POCUS exams produces a more effective tool for ocular pathologies in acute care settings and may be particularly valuable in resource-limited settings.

## INTRODUCTION

Point-of-care ultrasound (POCUS) plays a pivotal role in the evaluation of ocular complaints in the emergency department (ED). The rapid and non-invasive nature of ocular ultrasounds enables practitioners to assess the eye, regardless of periorbital swelling, making ocular ultrasound a safe and informative imaging modality.^1^ Eye complaints, including primary ophthalmologic pathology, infectious problems, and traumatic injuries, account for approximately 2–3% of all ED visits.^2^,^3^ A six-year analysis of eye-related ED visits found that 41.2% of ocular problems could be classified as emergent.^4^ Ocular complaints have a spectrum of severity, many of which require rapid diagnosis for appropriate treatment and recovery. Rapid diagnosis of retinal detachment (RD) is needed to prevent irreversible vision loss,^5^ whereas posterior vitreous detachment (PVD) is generally a benign condition.^6^

Ocular POCUS has been shown to accurately detect PVD, vitreous hemorrhage (VH), and RD in the ED setting,^3^,^7^–^14^ and previous research has shown emergency physicians (EP) to have high diagnostic accuracy with ocular POCUS. Blaivas et al performed a study (n=61) in a community ED with a residency program, which resulted in a sensitivity of 100% and specificity 97.2% in identifying a variety of ocular pathologies.^10^ Shinar et al found EPs at an academic center to have a sensitivity of 97% and a specificity of 92% in diagnosis of RD.^13^ Similarly, Yoonessi et al found academic EPs to have a sensitivity of 100% and a specificity of 83% in RD diagnosis with ocular POCUS.^11^ Baker et al found that academic EPs are “modestly accurate” at differentiating ocular diagnoses such as PVD (86% diagnostic accuracy) vs RD (74.6% diagnostic accuracy).^12^

While it has been established that POCUS can accurately detect these specific posterior vitreous pathologies, little is known as to whether over-gaining or under-gaining an ocular ultrasound image may ultimately result in erroneous diagnoses or missed abnormalities. One ophthalmologic report of B-scan ultrasonography use suggests that over-gaining an image can create a hyperechoic posterior vitreous humor; these artifactual internal echoes can result in false positives for RD or VH.^15^ However, this has not been evaluated using POCUS. Contrary to that, a protocol described by Gandhi et al notes that high-gain settings must be used to detect PVDs, while normal or low-gain settings are sufficient for RDs and VHs.^6^

We primarily investigate retrospectively how various gain levels impact the accuracy of ocular POCUS for detection and identification of any posterior chamber pathology. Secondarily, we focused specifically on RD, VH, and PVD to determine whether different gain levels impact the accuracy of detecting and identifying these specific pathologies. There are few studies that assess image optimization techniques and how they impact the overall accuracy of POCUS; establishing optimal gain settings for ocular ultrasound may improve ED diagnostic accuracy and efficiency by minimizing false positive and false negative diagnoses.

## METHODS

We performed a retrospective review of ED patients who received ocular POCUS examinations and ophthalmology consultations as part of their evaluation for eye complaints at our urban, Level I trauma center ED from November 2017–January 2021. We included adults aged 18 or older with a documented chief complaint of acute vision change if the following three conditions were met: 1) an ocular POCUS was documented via a “procedure note” in the electronic health record (EHR); 2) ophthalmology consultation was the gold standard for final diagnosis; and 3) there were stored images of the POCUS scan. Of the 706 patient charts that were accessed and reviewed, 383 met these characteristics. Exclusion criteria included incarcerated patients or those on a psychiatric hold. All research followed best practices of retrospective chart review as described by Worster and Bledsoe^16^ and was approved by the institutional review board at our institution.

**Population Health Research Capsule**

What do we already know about this issue?

*Point-of-care ultrasound can accurately identify ophthalmologic pathologies, including retinal detachment, vitreous hemorrhage, and posterior vitreous detachment*.

What was the research question?

*How do stratified gain levels impact the accuracy of detection of posterior chamber pathologies?*

What was the major finding of the study?

*High gain has increased sensitivity (91%, CI 82–97%) for detecting posterior chamber abnormalities compared to low gain (71%, CI 61–80%)*.

How does this improve population health?

*Incorporating high gain for ocular POCUS exams is an effective screening tool for detecting ocular pathologies in acute care and resource-limited settings*.

Ocular POCUS was performed by resident, fellow, and attending physicians. Any scans performed by residents were reviewed in real time by their supervising fellow or attending physician, which consisted of 53 fellows and attendings in total. All fellow and attending physicians were credentialed in interpretation of ocular ultrasound examinations and had performed at least 25 ocular scans in their own training or in the credentialing process. Every POCUS exam was performed with the Mindray TE7 ultrasound system (Mindray DS USA Inc., Mahwah, NJ) using the high-frequency linear probe. The gain values from 0–100 are displayed on the screen during use and were captured during the image/clip recording process. Gain is used for contrast resolution and is uniform amplification of the ultrasound signal that is returning to the transducer; therefore, it does not have any units of measurement. It is standardized across machines and brands, although auto-gain settings differ from brand to brand and differ based on probe and exam setting used. Auto-gain using the ocular setting on the Mindray TE7 is set to 48–55; however, clinicians do not always use the auto-gain setting.

We identified ocular US examinations through the billing reports provided by the coders. If ophthalmology consultation was obtained in the same visit, and the saved ocular US images could be reviewed, then that subject was eligible for enrollment. Chart reviewers were provided a standardized data collection form that was developed a priori (ED POCUS interpretation [PVD, VH, RD, normal], ophthalmology final diagnosis, and gain level used), and they were trained to collect necessary data points from the EHR. Chart reviewers then screened the obtained data for discrepancies, errors, or missing data points. Incomplete or erroneous ocular exams were excluded. Chart reviewers were blinded to the study endpoint. Original clinician interpretation of the US study documented in the EHR was used for ocular US exam findings. Two ultrasound fellows manually verified data.

The gain used by clinicians when scanning ranged from 29–100. Study participants were divided into three roughly equal groups by gain, and from this division we determined the stratified gain levels for analysis purposes as (25, 50] low gain; (50, 75] intermediate gain; and (75, 100] high gain. Each group had 147, 112, and 124 participants, respectively. A true positive or negative was operationally defined as a matching diagnosis that is detectable or undetectable through ocular POCUS (such as PVD, VH, RD, etc), respectively. We calculated sensitivity, specificity, positive predictive value (PPV), and negative predictive value (NPV) using the ophthalmologist’s final diagnosis as the gold standard. We analyzed data using STATA 17 (StataCorp LLC, College Station, TX), and we calculated test characteristics using “diagti” command.^17^ Continuous variables are reported as mean ± SD, and frequencies as N (%). Sensitivity and specificities are reported as point estimates (95% confidence interval).

## RESULTS

**Figure 1:**
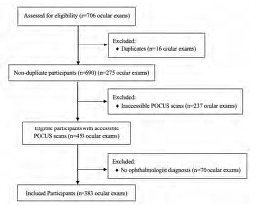
Flow chart of participants included (n= 383) and excluded (n=323) in the study. *POCUS*, point of care ultrasound.

A total of 706 records were accessed for this study. All duplicates (16) were removed. We excluded 237 patients due to the ocular ultrasound files having been corrupted or not saved. Lastly, 70 records did not have a final ocular diagnosis and were, therefore, excluded. We analyzed 383 (50.7%) charts that met inclusion criteria (Figure 1). The mean age was 49.2±15.8 years, and 207 (54.0%) were male. The right eye was affected in 187 patients (48.8%). Most common comorbidities included hypertension in 159 patients (41.5%) and diabetes in 132 patients (34.5%), while 48 (12.5%) had a history of glaucoma, 34 (8.9%) had prior RD, one (0.2%) had prior PVD, and there were zero patients (0%) with prior VH.

Per the ophthalmology final diagnoses, VH was the most diagnosed ocular pathology with a total of 84 cases (21.9%), followed by RD in 64 cases (16.7%), and PVD in 50 cases (13.1%). The total diagnoses amount to greater than 383 patients included in the study due to several patients having multiple ocular findings. Other final diagnoses included “no pathology noted,” or less common ocular pathologies, some of which are not always seen on POCUS: specifically, metamorphopsia; diabetic retinopathy; traumatic retinopathy; glaucoma; cataracts; vitreous degeneration; preretinal hemorrhage; papilledema; keratitis; conjunctivitis; iritis; optic nerve edema; macular holes; and macular edema.

**Figure 2:**
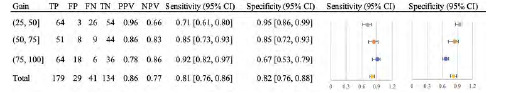
Diagnostic characteristics of emergency department ocular point-of-care ultrasound to detect any posterior chamber pathology. *TP*, true positive; *FP*, false positive; *FN*, false negative; *TN*, true negative; *PPV*, positive predictive value; *NPV*, negative predictive value; *CI*, confidence interval.

In our primary analysis we looked at the ability of EPs to detect any posterior chamber abnormality on ocular POCUS, and how the accuracy of detection changed at stratified gain levels (Figure 2). The images were found to have an overall sensitivity of 81% (76–86%), specificity of 82% (76–88%), PPV of 86% (81–91%), and NPV of 77% (70–83%). This was then further stratified by gain level, as shown in Figure 2. For the secondary analysis, we analyzed accuracy of detection of PVD, VH, and RD by EPs using ocular POCUS at stratified gain levels.

**Figure 3:**
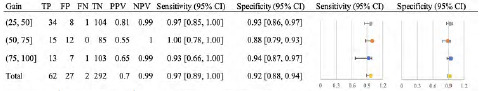
Diagnostic characteristics of emergency department ocular point-of-care ultrasound to detect retinal detachment. *TP*, true positive; *FP*, false positive; *FN*, false negative; *TN*, true negative; *PPV*, positive predictive value; *NPV*, negative predictive value; *CI*, confidence interval.

**Figure 4:**
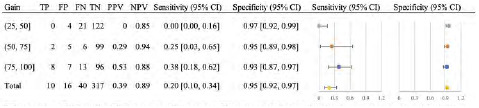
Diagnostic characteristics of emergency department ocular point-of-care ultrasound to detect posterior vitreous detachment. *TP*, true positive; *FP*, false positive; *FN*, false negative; *TN*, true negative; *PPV*, positive predictive value; *NPV*, negative predictive value; *CI*, confidence interval.

For the diagnosis of RD (Figure 3), there were 63 (16.4%) cases confirmed by ophthalmology gold standard. Overall, the images had a sensitivity of 97% (89–100%), specificity of 92% (88–94%), PPV of 70% (59–79%), and NPV of 99% (98–100%). This was then further stratified by gain level, as described in Figure 3. For the secondary analysis of PVD diagnosis (Figure 4), there were 47 (12.3%) cases confirmed by ophthalmology gold standard. Overall, the images had a sensitivity of 20% (10–34%), specificity of 95% (92–97%), PPV of 39% (20–59%), and NPV of 89% (85–92%). This was then further stratified by gain level, as described in Figure 4.

**Figure 5:**
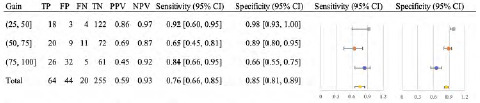
Diagnostic characteristics of emergency department ocular point-of-care ultrasound to detect vitreous hemorrhage. *TP*, true positive; *FP*, false positive; *FN*, false negative; *TN*, true negative; *PPV*, positive predictive value; *NPV*, negative predictive value; *CI*, confidence interval.

For the secondary analysis of VH diagnosis (Figure 5), there were 84 (21.9%) cases confirmed by ophthalmology gold standard. Overall, the images had a sensitivity of 76% (66–85%), specificity of 85% (81–89%), PPV of 59% (49–69%), and NPV of 93% (89–96%). This was then further stratified by gain level, as described in Figure 5.

## DISCUSSION

In this retrospective study, we aimed to investigate the accuracy of identification of posterior chamber pathologies at stratified gain levels. We found that increasing the gain for (or “overgaining”) ocular POCUS images allowed for increased sensitivity. The high-gain level (75, 100] was more sensitive than low-gain level (25, 50] for detecting these pathologies as confirmed by the gold standard ophthalmology consult (Figure 2). Higher sensitivity is preferable in ocular ultrasound; the cost of missing a case (ie, false negative), especially in the case of a RD, may result in vision loss, which would be life-altering for a patient, whereas the consequence of a false positive would result in potentially unnecessary specialist workup. Lower gains (26, 50] have the highest specificity for ruling in posterior chamber pathology but the greatest chance of missing pathology due to lower sensitivity. Therefore, when using ocular ultrasound as a screening modality, it is advantageous to incorporate higher gain levels.

Few previous studies have discussed that higher gain levels may be associated with increased false positive rates;^3^,^15^ however, high-gain levels have also been shown to be better for identifying posterior chamber abnormalities.^6^ Complementing our recommendation that high gains be incorporated into ocular POCUS exams, Shiner et al and Lahham et al. suggest that ultrasonographers should slowly adjust the ultrasound gain level while scanning to increase the likelihood of capturing pathology.^3^,^13^

Prior research has shown that the sensitivity of ocular POCUS in detecting a variety of ocular pathologies ranges from 97–100%, and that specificity ranges from 83–97.2%.^10^–^13^ For specific posterior chamber pathologies, high-gain settings have been recommended to detect PVDs, while normal or low-gain settings are sufficient for RDs and VHs.^6^ In our study, regardless of gain, ED ultrasonographers using POCUS perform well in the diagnosis of RD (sensitivity 97%) and are moderately accurate in diagnosing VH (sensitivity 76%).

In our secondary analysis, looking at stratified gain levels by specific pathology (RD, PVD, and VH), we found that sensitivities and specificities did vary depending on pathology. When stratified additionally into low-, intermediate-, and high-gain levels, our results for the detection of RD showed high sensitivity and specificity across all gain levels (Table 3), supporting the guidelines that low gains are sufficient to detect RD, and adding that high gains do not preclude accurate diagnoses. Due to its high sensitivity, POCUS can be considered a reliable screening tool for RD; therefore, ocular POCUS training in residencies should be promoted and incorporated into nearly all examinations for complaints of vision changes. As mentioned previously, the cost of missing a case, especially a RD, may be life-altering for a patient, whereas the consequence of a false positive would result in further specialist workup that later may turn out to be unnecessary. Lastly, since POCUS for RD has an acceptable specificity, it could serve as a reliable diagnostic tool to escalate to ophthalmological intervention, which may be beneficial in the outpatient setting or in underserved areas where ophthalmology is not readily available.

For PVD, there was a statistically significant increase in sensitivity at higher gain levels compared to lower gain levels; however, there were overall low sensitivities across all gain levels (Figure 4), suggesting that ocular POCUS may not be as effective at detecting PVD. It is possible that this is due to one of the following: 1) previous studies have noted that PVDs require higher gains; therefore, if PVDs are present along with other pathology, the ultrasonographer may have needed to increase to higher gains to catch the additional finding of PVD; or 2) there is a range of vitreous degeneration that may lead to PVD; however, the ophthalmologist’s diagnosis of vitreous degeneration was not counted as a PVD unless specifically stated. Specificity was high for all gain levels (93–97%); however, clinically this is of lower importance given that EPs are not typically screening specifically for PVDs, and these are often incidentally detected when looking for an intervenable pathology such as a RD.

Lastly, for VH, low gain had higher specificity compared to high gain (Figure 5). We did not find a difference in sensitivity of ocular US in detecting VH at different gain levels, which may reflect a limitation in sample size. Nevertheless, our results show that using low-gain levels as opposed to high-gain levels for suspected VHs minimize false positives.

Given the frequency of eye complaints seen in the ED^2^ and given that nearly half of them can be classified as emergent, the use of high gain on ocular POCUS exam can provide a manner to screen for these emergent cases to ensure that RDs that can be intervened upon are not missed. Most non-academic EDs do not have ophthalmologists readily available, making screening methods important tools for allocating limited resources. This could have particularly profound implications in resource-strained settings such as rural areas where ophthalmology consult may be miles away or nonexistent.

## LIMITATIONS

This study has several important limitations. A few studies have shown that the range and speed at which ultrasound gain levels are adjusted influence which pathologies are detected.^3^,^13^ Due to this study’s retrospective nature, the full range of gains that the physician ultrasonographer may have used were not obtained, and only when the user saved an image were we able to assess the gain level used for that image/clip. It is commonly taught and typical for users to increase the gain while performing ocular POCUS; therefore, our data may not have captured pathology that was noted by the EP at a lower gain but was only captured when saving images at a higher gain. If this is the case, sensitivity at lower gains would likely be improved and specificity would be decreased. Additionally, our study did not capture the length of time spent scanning each specific patient; it is worth considering whether patients with a higher pretest probability for concerning pathology were scanned for a longer duration of time and, therefore, were more likely to have had more accurate interpretations of their scans.

Another limitation is the potential for selection bias. Only those patients with an ocular ultrasound and an ophthalmology consult were included in the study. While this likely influenced the resulting test characteristics, this is also the population of greatest concern with a higher pretest probability for a RD or other concerning ocular pathology. The decision to perform an ocular ultrasound and consult ophthalmology represents the clinical judgment of the clinician, and ophthalmology consults are more likely to be obtained in more concerning cases; therefore, this was the population studied. Given that the ophthalmologic exam was used as the diagnostic gold standard, it is likely that patients with a low pretest probability were excluded.

Excluding patients with a low pretest probability either by not performing ocular POCUS or not consulting ophthalmology likely would result in an overestimation of the true sensitivity of ocular POCUS. Our results could be further corroborated by a prospective study design in which all patients presenting to the ED with an ocular concern receive an ophthalmologist consult, although this would be limited by institutional resources.

In addition, this study was conducted at an academic institution with an established emergency medicine residency and ultrasound fellowship programs, leading to a strong emphasis on ultrasound training and the use of ultrasound. All physician sonographers have had a baseline amount of training to perform and interpret ocular POCUS, and this may not be generalizable to all academic programs or private practices. Lastly, this study stratified the analysis by gain level, and further by disease (PVD, VH, RD) in the secondary analysis. This additional stratification led to smaller sample sizes in each specific disease-gain category, which may have affected the power of the analysis. As different pathology is seen at different gain levels,^6^,^13^ further study is warranted to corroborate optimal gain settings for each specific eye pathology.

## CONCLUSION

In the ED setting, high (75, 100] gain on ocular POCUS scanning has a higher degree of sensitivity for detecting any posterior chamber abnormality, as compared to lower (25, 50] gain levels. Thus, incorporating the use of high gain for ocular POCUS exams is an effective screening tool for ocular pathologies in acute care settings and may be particularly valuable in resource-limited settings.
